# Acetyl oxygen benzoate engeletin ester promotes KLF4 degradation leading to the attenuation of pulmonary fibrosis via inhibiting TGFβ1–smad/p38MAPK–lnc865/lnc556–miR-29b-2-5p–STAT3 signal pathway

**DOI:** 10.18632/aging.202975

**Published:** 2021-04-30

**Authors:** Ke Shen, Ruiqiong Li, Xiaoli Zhang, Guiwu Qu, Rongrong Li, Youlei Wang, Bo Liu, Changjun Lv, Minge Li, Xiaodong Song

**Affiliations:** 1 Department of Cellular and Genetic Medicine, School of Pharmaceutical Sciences, Binzhou Medical University, Yantai 264003, China; 2 School of Nursing, Binzhou Medical University, Yantai 264003, China; 3 Department of Respiratory Medicine, Binzhou Medical University Hospital, Binzhou Medical University, Binzhou 256602, China

**Keywords:** pulmonary fibrosis, lncRNA, miRNA, STAT3, KLF4

## Abstract

Pulmonary fibrosis is a common pulmonary interstitial disease of pathogenesis without effective drugs for treatment. Therefore, discovering new and effective drugs is urgently needed. In the present study, we prepared a novel compound named acetyl oxygen benzoate engeletin ester (AOBEE), investigated its effect on experimental pulmonary fibrosis, and proposed a long non-coding RNA (lncRNA)-mediated mechanism of its action. Bleomycin-induced pulmonary fibrosis in mice exhibited that AOBEE improved forced vital capacity (FVC) and alveolar structure and inhibited α-SMA, vimentin, and collagen expression. TGFβ1-stimulated fibroblast L929 cells showed that AOBEE reduced these fibrotic proteins expression and inhibited the activated-fibroblast proliferation and migration. Whole transcriptome sequencing was performed to screen out lncRNA-lnc865 and lnc556 with high expression under bleomycin treatment, but AOBEE caused a considerable decrease in lnc865 and lnc556. Mechanistic study elucidated that AOBEE alleviated pulmonary fibrosis through lnc865- and lnc556-mediated mechanism, in which both lnc865 and lnc556 sponged miR-29b-2-5p to target signal transducer and activator of transcription 3 (STAT3). Further signal pathway inhibitors and the Cignal Finder 45-pathway reporter array illustrated that the up- and downstream pathways were TGFβ1–smad2/3 and p38MAPK, and Krüppel-like factor 4 (KLF4), respectively. In conclusion, AOBEE promoted KLF4 degradation leading to the attenuation of pulmonary fibrosis by inhibiting TGFβ1–smad/p38MAPK–lnc865/lnc556–miR-29b-2-5p–STAT3 signal pathway. We hope this work will provide valuable information to design new drugs and therapeutic targets of lncRNAs for pulmonary fibrosis treatment.

## INTRODUCTION

Pulmonary fibrosis is a common pulmonary interstitial disease of pathogenesis, with progressively increased dyspnea as the most prominent clinical manifestation [1]. Among them, pulmonary fibrosis with unknown cause is called idiopathic pulmonary fibrosis (IPF). The patient median survival time is only 3–5 years [2]. Age is a main risk factor of pulmonary fibrosis. Most of the patients are over 60 years old. With the rising aging population, the incidence of pulmonary fibrosis is increasing. Clinical studies have reported that bifenidone and nintedanib can improve the symptoms of pulmonary fibrosis by reducing the maximum forced vital capacity (FVC), but the low drug tolerance and adverse side effects limit their use [3, 4]. Currently, lung transplantation is the only therapeutic option for pulmonary fibrosis. Thus, exploring the molecular mechanism of pathogenesis and finding effective drugs for treating pulmonary fibrosis are urgently needed.

Long non-coding RNA (lncRNA) is a type of regulatory non-coding RNA with more than 200 nucleotides in length that cannot encode protein. Through various mechanisms, lncRNAs have been implicated in numerous cellular processes, such as cell cycle, death, proliferation, differentiation and so on. As a result, more and more lncRNAs have been involved in various human diseases [5]. Now, lncRNAs have become the targets of gene therapy and drug action. For example, lncITPF forms RNA–protein complex with hnRNP-L to accelerate pulmonary fibrosis through the TGFβ1–smad2/3 signal pathway. Interfering lncITPF expression can attenuate pulmonary fibrosis [6]. Astaxanthin exerts its anti-pulmonary fibrosis effect by downregulating lncITPF expression [7]. LncH19 gene therapy prevents and reverses experimental pressure-overload-induced heart failure [8]. *Angelica sinensis* polysaccharide, extracted from the roots of *angelica*, suppresses pulmonary fibrosis via downregulating lncDANCR-targeted FOXO3 protein levels in an AUF1-dependent manner [9]. Astragaloside IV (ASV), a bioactive saponin extracted of Astragalus root, increases lncSIRT1 AS to exert its anti-fibrotic effect on idiopathic pulmonary fibrosis [10]. On the whole, few studies have focused on lncRNA as the target of drug action in pulmonary fibrosis.

**Figure 1:**
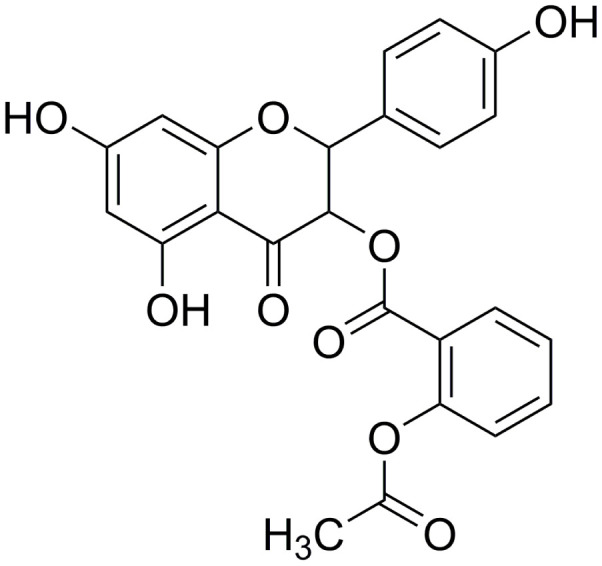
Molecular formula of AOBEE.

In our previous study, we found that engeletin has anti-pulmonary fibrosis function by inhibiting lnc949 to respond to endoplasmic reticulum stress [11]. However, engeletin has poor water solubility. And it also can be easily oxidized and discolored. Therefore, our team conducted a series of optimized structural modifications to prepare a new compound, and we named it as acetyl oxygen benzoate engeletin ester (AOBEE), which has stronger activity and better stability and water solubility than engeletin (Figure 1). In the present study, we investigated the anti-fibrotic effect of AOBEE in TGFβ1-stimulated lung fibroblast and bleomycin (BLM)-treated mice. Differently expressed lncRNAs (lnc865 and lnc556) were further screened by RNA sequencing under the action of AOBEE. Mechanistically, AOBEE promoted Krüppel-like factor 4 (KLF4) degradation by inhibiting the lnc865/lnc556–miR-29b-2-5p–STAT3 axis through the TGFβ1–smad2/3 and p38MAPK signaling pathways.

## RESULTS

### Anti-pulmonary fibrosis effect of AOBEE *in vivo* and *in vitro*

**Figure 2:**
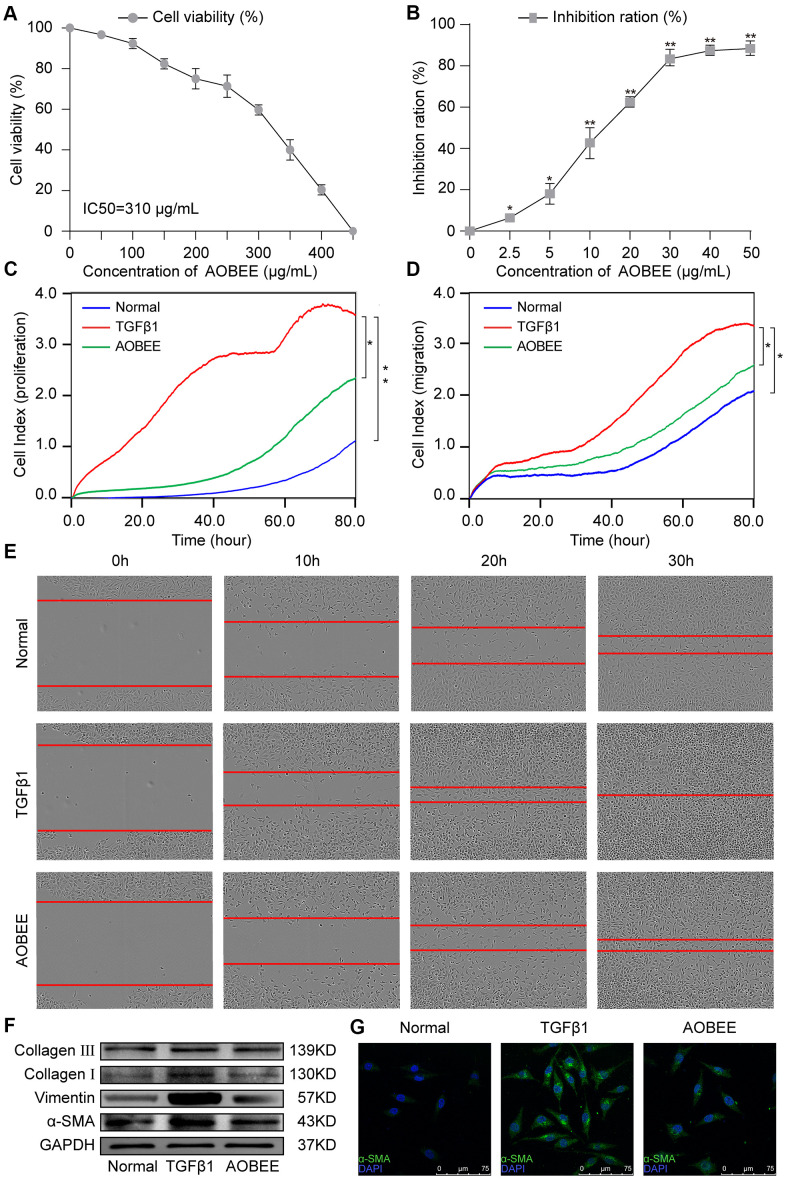
**AOBEE cytotoxicity and anti-pulmonary fibrosis *in vitro*.** (**A**) CCK-8 was used to test AOBEE toxicity in normal L929 cells. IC50 was approximately 310 μg/mL. (**B**) Inhibition of AOBEE on TGFβ1-treated cells in a dose-dependent manner. AOBEE had a significant inhibitory effect at a concentration of 10 μg/mL. (**C**) L929 cells were first administered with 5 ng/mL of TGFβ1 for 72 h and then cotreated with 24 μg/mL AOBEE. Using an xCELLigence RTCA instrument, the proliferation curves showed that AOBEE inhibited the activated-fibroblast proliferation compared with those in the TGFβ1 treatment group. (**D**) The migration curves revealed that AOBEE significantly repressed the activated-fibroblast migration compared with those in the TGFβ1 treatment group. (**E**) Images automatically monitored by an IncuCyte S3 instrument confirmed that AOBEE slowed the migration of activated fibroblasts at different time points. (**F**) AOBEE substantially reduced the expression of related fibrotic proteins including α-SMA, vimentin, and collagen I and III. (**G**) The immunofluorescence images showed that the TGFβ1-treated fibroblasts were spindle-shaped and had increased α-SMA. AOBEE improved cell state and reduced α-SMA expression. Each bar represents the mean ± SD (n = 6; *p < 0.05, **p < 0.01).

First, AOBEE toxicity was evaluated *in vitro* by using cell counting kit-8 assay (CCK-8). The mouse fibroblast L929 cells were incubated for 48 h under increasing concentrations of AOBEE. As compared with untreated cells, the half maximal inhibitory concentration (IC50) was 310 μg/mL (Figure 2A). To better understand the anti-fibrotic mechanism of AOBEE, 5 ng/mL of TGFβ1-activated mouse fibroblast L929 cells were selected to establish a pulmonary fibrosis cell model. Then, the inhibitory effects of AOBEE on L929 cells treated with TGFβ1 for 72 h were tested. AOBEE inhibited cell viability in a dose-dependent manner when the cells were exposed to graded doses (2.5–50 μg/mL) for 48 h. The most pronounced inhibition was at the beginning of 10 μg/mL (Figure 2B). After 30 μg/mL, the inhibition rate did not increase significantly. So 24 μg/mL concentration was used for further *in vitro* studies. Real-time cellular analysis (RTCA) experiments were performed to monitor the proliferation and migration of activated L929 cells. The curves demonstrated that AOBEE inhibited activated fibroblast proliferation and migration compared with TGFβ1 treatment alone (Figure 2C, 2D). The wound healing results confirmed that AOBEE repressed the migration of activated fibroblast (Figure 2E). The change of fibroblast shape and reduction of collagen deposition, α-SMA, and vimentin indicated that AOBEE had an obvious anti-pulmonary fibrosis effect by blocking the proliferation and migration of the activated fibroblasts (Figure 2F, 2G).

**Figure 3:**
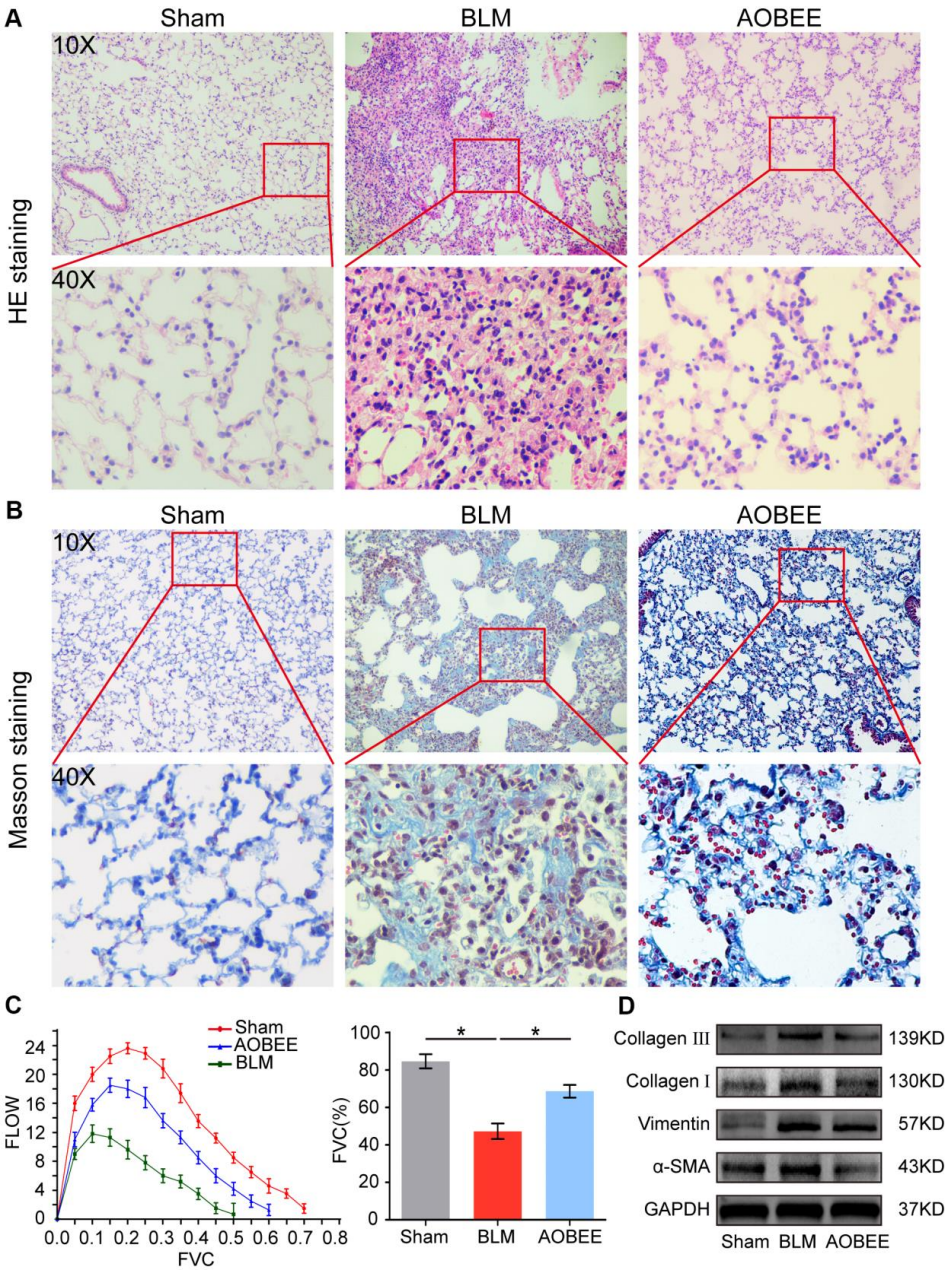
**Anti-pulmonary fibrosis of AOBEE *in vivo*.** (**A**) HE staining showed that AOBEE improved the alveolar structure of mice, such as more spacious alveolar space and thinner alveolar walls compared with those in the BLM group. (**B**) Masson staining showed that AOBEE treatment reduced the deposition of collagen fibers. (**C**) The Buxco PFT analysis system showed that AOBEE enhanced the pulmonary function of mice compared with those in the BLM group. (**D**) Western blot indicated that AOBEE inhibited α-SMA, vimentin, and collagen expression levels. Each bar represents the mean ± SD, n = 6, *p < 0.05.

Then, the anti-pulmonary fibrosis effect of AOBEE was further explored *in vivo* by using Hematoxylin eosin (HE) and Masson staining, pulmonary function and fibrotic marker proteins in a bleomycin (BLM)-induced mouse model. The results of HE staining showed that the alveolar structure in the BLM group mice were damaged and accompanied by slight inflammatory cell infiltration. AOBEE treatment improved the symptoms and alveolar structure (Figure 3A). Masson staining demonstrated that AOBEE significantly reduced collagen fibers compared with those in the BLM-induced group (Figure 3B). Meanwhile, AOBEE treatment obviously promoted FVC and pulmonary compliance in mice (Figure 3C) and induced a considerable decrease in fibrotic marker proteins collagen I and III, vimentin, and α-SMA (Figure 3D). All the above findings indicated that AOBEE had an anti-pulmonary fibrosis effect *in vivo*.

### Lnc865 and lnc556 mediated the anti-pulmonary fibrosis effect of AOBEE

**Figure 4:**
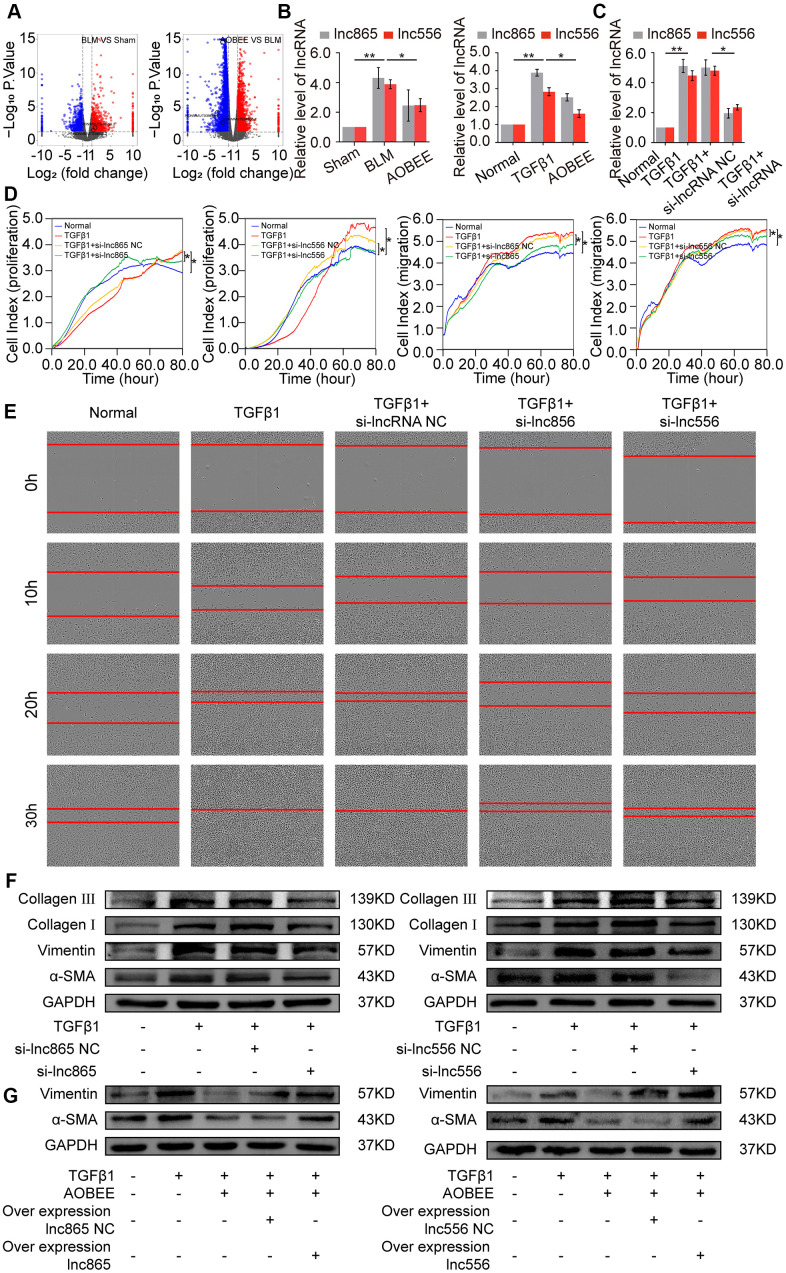
**Inhibition of AOBEE on lnc865 and lnc556.** (**A**) RNA sequencing demonstrated the differently expressed RNA transcriptions in BLM group compared with those in sham group (left), and in AOBEE + BLM group compared with those in BLM group (right). Lnc865 and lnc556 upregulated in BLM group compared with those in sham group, and downregulated in AOBEE + BLM group compared with those in BLM group. (**B**) qRT-PCR confirmed that BLM caused a marked increase of lnc865 and lnc556 expression, whereas AOBEE caused a marked decrease of their expression in BLM-treated mice and TGFβ1-induced L929 cells. (**C**) The efficacy of knockdown with si-lnc865 and si-lnc556 was examined through qRT-PCR. (**D**) The real-time cell proliferation and migration experiments indicated that si-lnc865 and si-lnc556 inhibited the proliferation and migration of cells activated by TGFβ1. (**E**) Images automatically monitored by an IncuCyte S3 instrument showed that si-lnc865 and si-lnc556 repressed the migration of L929 cells activated by TGFβ1 at different time points. (**F**) Expression levels of collagen, vimentin, and α-SMA were downregulated in the si-lnc865 and si-lnc556 groups compared with the TGFβ1-treated group. (**G**) The results of rescue experiment showed that AOBEE treatment reduced the expression of α-SMA, vimentin. Overexpression of lnc865 and lnc556 reversed this treatment effect. Each bar represents the mean ± SD, n = 6; *p < 0.05, **p < 0.01.

To further explore which lncRNAs participate in the anti-pulmonary fibrotic regulation of AOBEE, whole transcriptome sequencing was performed to analyze the changes of RNA transcriptions in sham group, BLM group and AOBEE treatment group. Among the numerous altered lncRNAs, lnc865 and lnc556 were upregulated in the BLM-treated group compared with the sham group. Both were downregulated after AOBEE treatment (Figure 4A). Because lnc865 and lnc556 were the focus of our previous study [12], they were selected for follow-up studies. Quantitative real-time PCR (qRT-PCR) was conducted to confirm the sequencing results *in vivo* and *in vitro* (Figure 4B).

Next, small interfering RNAs of lnc865 and lnc556 (si-lnc865 and si-lnc556) were designed to investigate the function of lnc865 and lnc556 under AOBEE treatment. After transfection with si-lnc865 and si-lnc556, the expression level of lnc865 and lnc556 was significantly downregulated compared with the control group (Figure 4C), indicating that interference was effective. The real-time cell proliferation and migration curves showed that the knockdown of lnc865 and lnc556 inhibited the proliferation and migration of cells activated by TGFβ1 (Figure 4D). The wound healing assay confirmed these results (Figure 4E). Meanwhile, the expression levels of fibrotic marker proteins, such as collagen, vimentin, and α-SMA, were downregulated in the si-lnc865 and si-lnc556 groups (Figure 4F). The above findings suggested that lnc865 and lnc556 increased the development of pulmonary fibrosis, and AOBEE alleviated the pulmonary fibrosis process by inhibiting lnc865- and lnc556-mediated signal pathways. The rescue experiments were further designed to verify that the treatment of AOBEE depended on lnc865 and lnc556. The results showed that AOBEE reduced the expression of α-SMA, vimentin. Whereas, both lnc865 and lnc556 overexpression reversed AOBEE treatment effect (Figure 4G).

### AOBEE attenuated pulmonary fibrosis by inhibiting the lnc865/lnc556–miR-29b-2-5p–STAT3 axis

**Figure 5:**
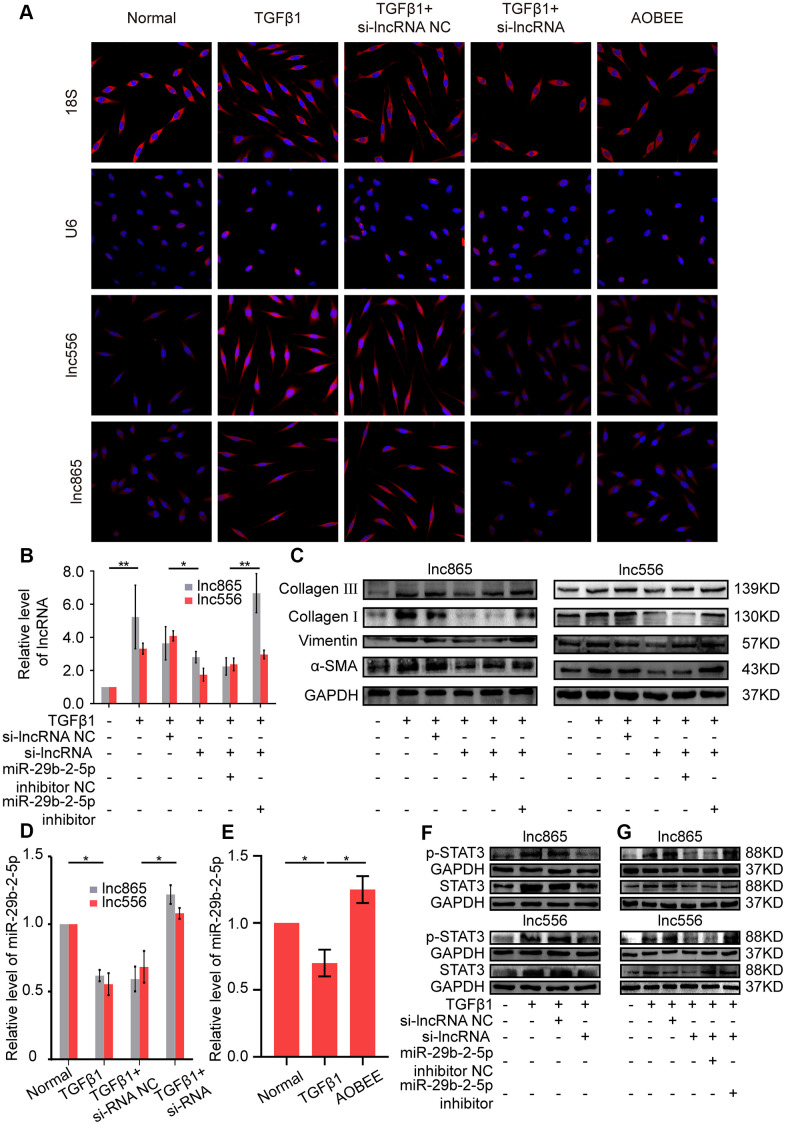
**AOBEE attenuated pulmonary fibrosis by blocking the lnc865/lnc556–miR-29b-2-5p–STAT3 axis.** (**A**) RNA-FISH showed that lnc865 and lnc556 mainly existed in the cytoplasm, and AOBEE induced a decrease of their expression but did not cause their translocation. (**B**) The rescue experiments reflected that si-lnc865 and si-lnc556 decreased lnc865 and lnc556 expression, but the miR-29b-2-5p inhibitor increased their expression. (**C**) The rescue experiments showed that si-lnc865 and si-lnc556 induced a substantial decrease of vimentin, α-SMA, and collagen I and III, but the miR-29b-2-5p inhibitor induced a substantial increase of the expression of these proteins. (**D**) RNA interference on lnc865 and lnc556 increased miR-29b-2-5p expression. (**E**) AOBEE promoted miR-29b-2-5p expression. (**F**) Western blot revealed that si-lnc865 and si-lnc556 decreased STAT3 and p-STAT3 expression. (**G**) The rescue experiments demonstrated that the miR-29b-2-5p inhibitor reversed the effects of si-lnc865 and si-lnc556. Each bar represents the mean ± SD, n = 6; *p < 0.05, **p < 0.01.

Gene location determines the regulatory pattern of genes, such as transcriptional regulation or post-transcriptional regulation. Single-molecule RNA fluorescence *in situ* hybridization (FISH) results exhibited that lnc865 and lnc556 were mainly enriched in the cytoplasm, indicating that they exercised post-transcriptional regulation on their target genes. Meanwhile, AOBEE treatment caused a considerable decrease in lnc865 and lnc556 expression but did not induce their translocation (Figure 5A). The common post-transcriptional pattern of lncRNA is competing endogenous RNA in the cytoplasm, in which lncRNA has the same miRNA response elements (MREs) like mRNA, and competes with mRNA to bind miRNA. Through this sponge function, lncRNA inhibits or activates the targeted mRNA [13]. Our previous study confirmed that miR-29b-2-5p is one of the target miRNAs for lnc865 and lnc556, and it also can be binded by signal transducer and activator of transcription 3 (STAT3) [12]. Thus, the regulatory role of miR-29b-2-5p was investigated under AOBEE action. The rescue experiments validated that RNA interference of lnc865 and lnc556 caused an obvious decrease of lnc865, lnc556, and fibrotic proteins including vimentin, α-SMA, and collagen I and III; however, the miR-29b-2-5p inhibitor reversed their downward trend, indicating that the pro-fibrotic function of lnc865 and lnc556 depended on miR-29b-2-5p (Figure 5B, 5C). After RNA interference on lnc865 and lnc556, miR-29b-2-5p increased (Figure 5D). Further qRT-PCR results demonstrated that AOBEE treatment enhanced miR-29b-2-5p expression (Figure 5E). RNA interference on lnc865 and lnc556 reduced STAT3 and p-STAT3 expression (Figure 5F). And the rescue experiments further elucidated that the miR-29b-2-5p inhibitor reversed the downward trend of STAT3 and p-STAT3 (Figure 5G), which indicated that the inhibition of si-lncRNA on STAT3 depended on miR-29b-2-5p. All the above findings revealed that AOBEE attenuated pulmonary fibrosis by inhibiting the lnc865/lnc556–miR-29b-2-5p–STAT3 axis.

### Regulatory up- and downstream signal pathways of lnc865/lnc556–miR-29b-2-5p–STAT3 axis under AOBEE treatment

**Figure 6:**
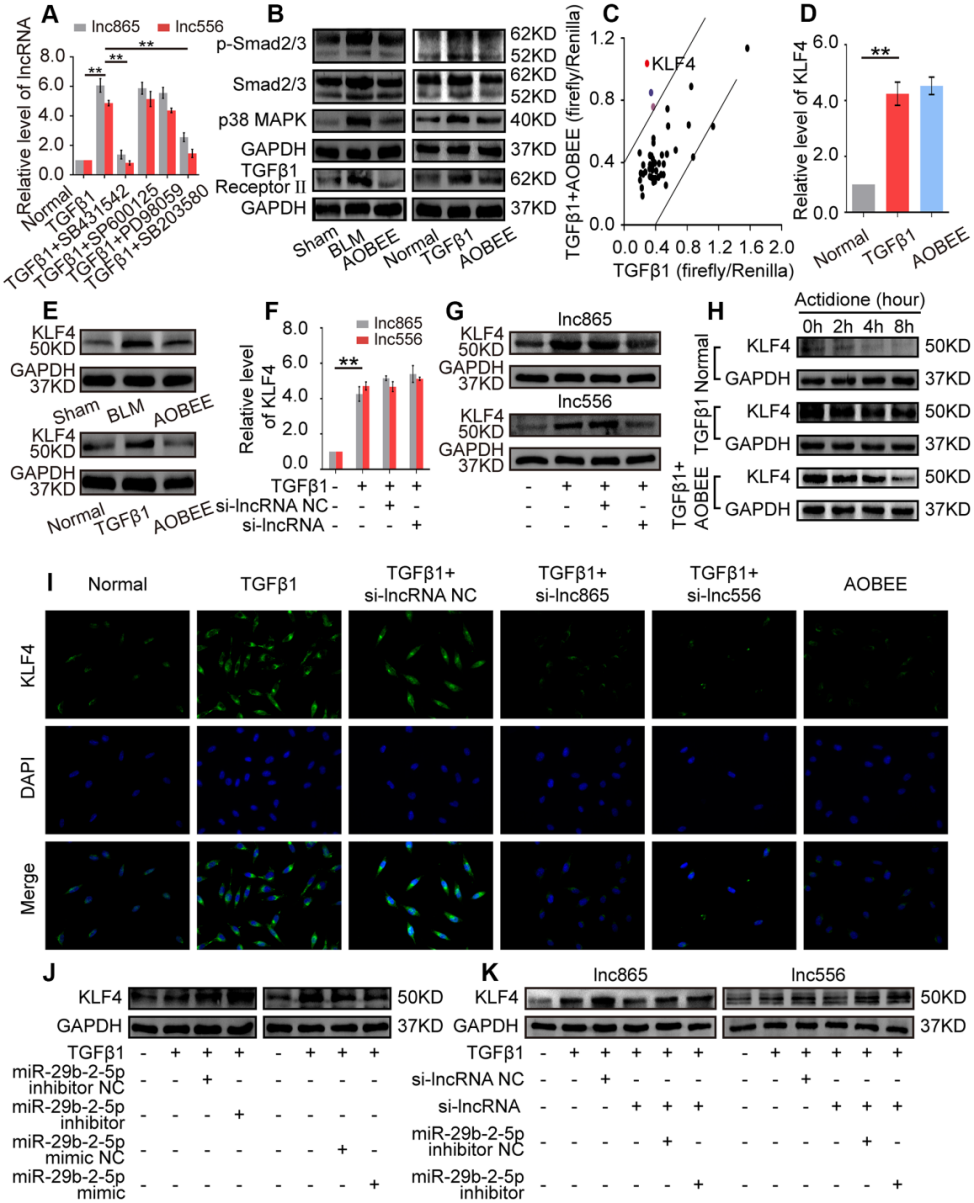
**Exploration of the up- and downstream signal pathways regulated by AOBEE.** (**A**) Signal pathway inhibitors were used to detect the changes of lnc865 and lnc556 expression. SB431542 and SB203580 inhibited lnc865 and lnc556 expression. (**B**) Western blot showed that AOBEE decreased p38MAPK, smad2/3 and TGFβ1 receptor II expression in *in vivo* and *in vitro* models. (**C**) Identification of downstream signal pathways affected by AOBEE in L929 cells. The x- and y-axes represent the normalized ratio of firefly/Renilla luciferase activities, respectively. KLF4 is one of the significant changes of signal pathways. (**D**) AOBEE did not cause any changes of KLF4 expression at the mRNA level by using qRT-PCR. (**E**) Western blot showed that AOBEE repressed KLF4 expression at the protein level. (**F**) Si-lnc865 and si-lnc556 did not cause any changes of KLF4 expression at the mRNA levels by using qRT-PCR. (**G**) Western blot showed that si-lnc865 and si-lnc556 repressed KLF4 expression at the protein level. (**H**) The degradation of KLF4 was detected by using the protein synthesis inhibitor actidione in L929 cells. The fastest degradation rate was in the normal group and the slowest degradation rate was in the TGFβ1 group. AOBEE promoted KLF4 degradation compared with TGFβ1. (**I**) Immunofluorescence experiments verified that AOBEE, si-lnc865, and si-lnc556 inhibited KLF4 expression at the protein level. (**J**) MiR-29b-2-5p inhibitor increased KLF4 expression and miR-29b-2-5p mimic decreased KLF4 expression. (**K**) The rescue experiments showed that si-lnc865 and si-lnc556 decreased KLF4 expression, and miR-29b-2-5p inhibitor increased KLF4 expression at the protein level. Each bar represents the mean ± SD, n = 6; *p < 0.05, **p < 0.01.

To further elucidate the signaling pathway by which AOBEE alleviates pulmonary fibrosis, we evaluated the up- and downstream pathways regulated by AOBEE. First, the upstream pathway was explored by using signal pathway inhibitors, namely, SB431542, SB203580, SP600125, and PD98059. They are specific inhibitors to the TGFβ1–smad2/3, p38MAPK, JNK, and ERK signaling pathways, respectively. SB431542 and SB203580 blocked the expression of lnc865 and lnc556, indicating that their upstream signal pathways were TGFβ1–smad2/3 and p38MAPK (Figure 6A). Western blot confirmed that AOBEE inhibited p38MAPK, smad2/3 and TGFβ1 receptor II expression (Figure 6B), which suggested that lnc865 and lnc556 mediated the anti-pulmonary fibrosis effect of AOBEE through the TGFβ1–smad2/3 and p38MAPK signaling pathways.

Then, the downstream pathway was detected by using the Cignal Finder 45-pathway reporter array. The results showed that AOBEE strongly repressed the luciferase activities of KLF4 reporter gene compared with those of other reporter genes (Figure 6C). qRT-PCR indicated that AOBEE had no effect on KLF4 at the mRNA level (Figure 6D), but Western blot results demonstrated that AOBEE inhibited KLF4 expression at the protein level *in vivo* and *in vitro* (Figure 6E). Si-lnc865 and si-lnc556 also had no effect on KLF4 at the mRNA level (Figure 6F), but the Western blot results demonstrated that si-lnc865 and si-lnc556 inhibited KLF4 expression at the protein level (Figure 6G). To clarify this finding, actidione, a protein synthesis inhibitor, was used to detect the degradation of KLF4 under AOBEE treatment. The degradation rate was the fastest in the normal group and the slowest in the TGFβ1 group. The results showed that TGFβ1 inhibited KLF4 degradation. Whereas, AOBEE promoted KLF4 degradation compared with TGFβ1 (Figure 6H). Immunofluorescence experiments further confirmed that AOBEE, si-lnc865, and si-lnc556 reduced KLF4 expression at the protein level (Figure 6I). To infer, AOBEE may decrease the translation of KLF4, not its transcription, as well as lnc865 and lnc556. The miR-29b-2-5p inhibitor increased KLF4 expression, but the miR-29b-2-5p mimic decreased KLF4 expression (Figure 6J). The rescue experiments identified that si-lnc865 and si-lnc556 prevented KLF4 expression at the protein level, and the miR-29b-2-5p inhibitor reversed KLF4 expression (Figure 6K). All the above findings indicated that AOBEE prevented pulmonary fibrosis through the lnc865/lnc556 mediated downstream pathway of KLF4.

## DISCUSSION

**Figure 7:**
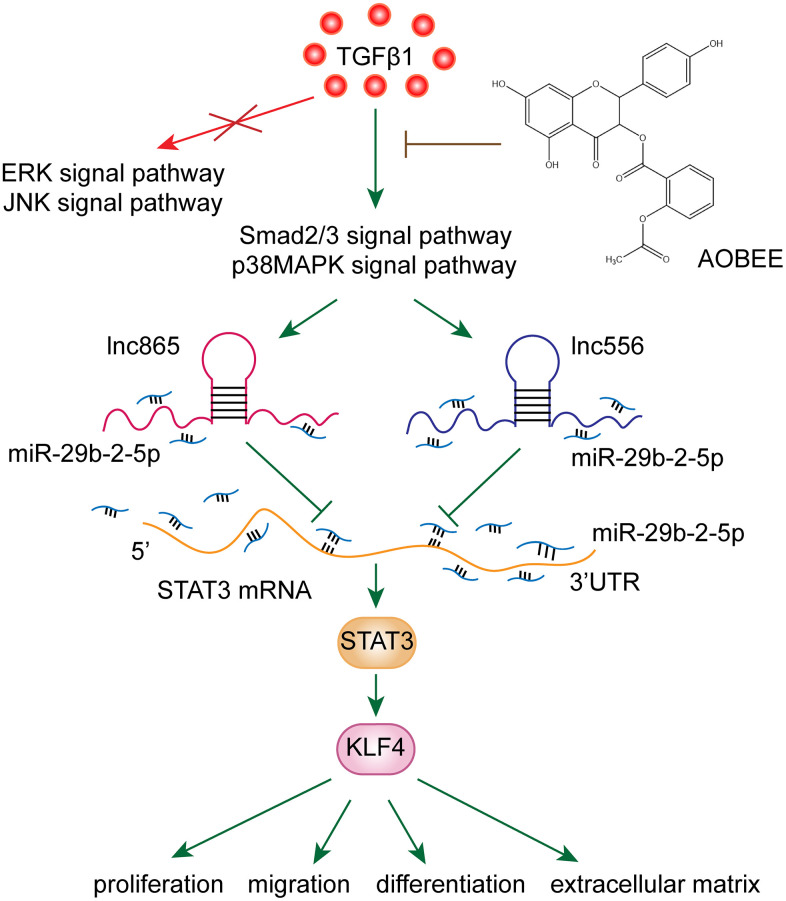
AOBEE reduced the expression of KLF4 by inhibiting the lnc865/lnc556–miR-29b-2-5p–STAT3 axis via the TGFβ1–smad2/3 and p38MAPK signaling pathways.

To date, various mechanisms have been developed for pulmonary fibrosis, and the irreversible transformation of fibroblasts into myofibroblasts is a noteworthy mechanism of fibrosis [14]. After fibroblasts differentiate into myofibroblasts, myofibroblasts exhibit uncontrolled proliferation and migration characteristics, which have been accompanied by collagen fiber deposition [15]. The deposition of collagen in the alveoli alleviates the function of alveolar gas exchange, and fibrosis lesions form at the deposit. Therefore, blocking fibroblast-to-myofibroblast transition is a therapeutic strategy to attenuate pulmonary fibrosis. Our results showed that AOBEE reduced the expression of KLF4 by inhibiting the lnc865/lnc556–miR-29b-2-5p–STAT3 axis via the smad2/3 and p38MAPK signaling pathways, thereby blocking fibroblast-to-myofibroblast differentiation, myofibroblast proliferation and migration, and extracellular matrix deposition (Figure 7).

LncRNAs are critical regulators in the pathogenesis by serving as promoters or suppressors in various diseases. Therefore, an increasing number of studies are starting to explore their clinical application, including disease markers and therapeutic targets for gene and drug therapies [16, 17]. LncGAS5 knockdown leads to anti-renal fibrosis via competitively binding miR-96-5p, which targets to repress fibronectin expression [18]. LncMT1DP in the blood is a potential marker for cadmium-induced nephrotoxicity. Mechanistically, liver-derived exosome-laden lncMT1DP indirectly breaks the equilibrium between the pro-apoptotic and anti-apoptotic effects conducted by BAX and Bcl-xL, respectively [19]. Circulating lncSNHG11 may be a novel biomarker for the early diagnosis and prognosis of colorectal cancer, which can enhance tumor cell proliferation and metastasis through the Hippo signal pathway [20]. Undoubtedly, identifying lncRNAs as novel therapeutic targets will lead to drug discovery [21]. An lncRNA–mRNA-weighted co-expression network analysis demonstrated the mechanism of Caulophyllum robustum Maxim against rheumatoid arthritis through lncRNA-mediated signal pathways including TNF, Toll-like receptor, and chemokine signaling pathways [22]. Wei et al. discovered that 10 lncRNAs, NONRATT009275.2, NONRATT025409.2, NONRATT025419.2, MSTRG.7681.1, ENSRNOT00000084373, NONRATT000512.2, NONRATT006734.2, ENSRNOT00000084386, NONRATT021738.2, and ENSRNOT00000084080, are involved in the treatment of chronic glomerulonephritis by Qi Teng Xiao Zhuo granules [23]. However, most of these studies only screened the differentially expressed lncRNAs under drug action. Studies on lncRNA-mediated drug mechanism are very limited. This work clarified that lnc865 and lnc556 targeted the same gene STAT3 via sponging miR-29b-2-5p to participate in the regulatory mechanism of AOBEE treatment. The upstream was TGFβ1–smad and p38MAPK signaling pathways, and the downstream was KLF4 signaling pathway. Wang et al. reported that KLF4 can activate the TGFβ1–smad signaling pathway [24], which supports our finding.

Interestingly enough, KLF4 has different functions during fibrogenesis. KLF4 is a zinc finger transcription factor that can accelerate wound healing [25]. Experiments on conditional mutant mice lacking KLF4 or TNFα proved that macrophage KLF4 attenuates TNFα-mediated kidney injury and fibrosis [26]. KLF4 may be the therapeutic target of pulmonary fibrosis by inhibiting the differentiation of lung resident mesenchymal stem cells [24]. However, KLF4 is upregulated in fibrotic skin and lungs from mice with systemic sclerosis. Leflunomide prevents ROS-induced systemic fibrosis by downregulating KLF4 signal pathway [27]. Our findings elucidated that KLF4 was highly expressed in BLM-treated mice and TGFβ1-induced L929 cells. It is the downstream signaling pathway under AOBEE action. STAT3, a transcription factor, is elevated in the lungs of IPF patients and triggers the expression of various fibrotic genes [28]. KLF4 is one of the important target genes of STAT3 [29]. Through STAT3 regulation, KLF4 takes part in cell fate determination [30], cell differentiation [29], and tumorigenesis [31]. This study revealed that AOBEE repressed KLF4 through the regulatory network of lnc865/lnc556–miR-29b-2-5p–STAT3, blocking lung fibrogenesis.

Collectively, the novel compound AOBEE prepared by our team exhibited marked anti-fibrotic effects *in vivo* and *in vitro*. Our study provides valuable information in the design of new drugs and presents candidate therapeutic targets for drug treatment.

## MATERIALS AND METHODS

### Animal model and ethics statement

The C57BL/6 mice with an average body weight of 20 ± 5 g were purchased from the Experimental Animal Breeding Company of Jinan Pengyue Laboratory (Jinan, China). Animal experiments were conducted according to the guidance of the Animal Experiment Ethics Committee of Binzhou Medical University. The mice were divided into 3 groups: sham group, BLM group and AOBEE + BLM group (10 mice per group), and 5 mg/kg BLM dissolved in saline was sprayed into the mice lung to induce pulmonary fibrosis by using a microsprayer (Penn-Century, Inc., USA). The sham group only received the same amount of saline as the control group. On 14^th^ day, 25 mg/kg AOBEE was injected intraperitoneally every day. On the 28^th^ day, all mice were sacrificed. Lung tissues were collected and frozen immediately in liquid nitrogen for further study.

### Cell culture and treatment

Mouse lung fibroblast L929 cell line was purchased from the cell bank of the Chinese Academy of Sciences. Cells were cultured in modified MEM medium containing 10% newborn calf serum, 100 U/mL penicillin and 100 mg/mL streptomycin, and incubated at 37° C under 5% CO_2_. According to experimental requirement, 5 ng/mL TGFβ1 was added to the cells for 72 h, then the cells were treated with 24 μg/mL AOBEE for 48 h. si-lnc865 and si-lnc556, and negative control (si-lnc865/556 NC) were transfected to L929 cells with Ribo FECTTM CP transfection kit (Ribo Bio Company, Guangzhou, China).

### Pulmonary function analysis

The mice pulmonary function was tested by Buxco pulmonary function testing system (DSI Buxco, USA) in accordance with the manufacturer’s instructions. In brief, the instrument was first adjusted to zero and set the parameters. Then the mice were put into the detection device and detected FVC with FinePointe PFT software and the Buxco PFT Controller hardware.

### Wound healing assay

5 × 10^5^/mL L929 cells were seeded in a 96-well plate. The cells were wounded with scratcher and placed in IncuCyte S3 (Essen BioScience, USA) for real-time dynamic observation. Images were taken on IncuCyte S3 software.

### Real-time cellular proliferation and migration analyses

The cells were cultured in E-plate and CIM-plate, respectively. In the migration plate, 5×10^4^ cells were seeded in a basal medium containing 1% fetal bovine serum in the upper chamber. The lower chamber contained a complete medium with growth factors and additives. The instrument software calculated the number of proliferating and migrating cells. Cell proliferation and migration were monitored and automatically recorded by using real-time cellular analysis instrument (ACEA Biosciences, Inc., Hangzhou, China).

### RNA FISH

FISH detection was performed using Ribo™ FISH kit (Ribobio, China. Products numbers as the follows: lnc556: lnc1101365; lnc865: lnc1101366). The lnc865/556 fish probe, 18s and U6 probe labeled with Cy3 fluorescent dye were designed and synthesized by Ribobio company. The cells were fixed in 4% paraformaldehyde, permeabilized for 3 minutes with PBS solution containing 0.5% Triton X-100, and pre-treat with pre-hybridization solution. Then the cells and probe were mixed at 4° C overnight. After hybridization, the nuclei were stained with DAPI for 10 minutes. The images were observed and analyzed with a confocal microscope (Leica, Germany).

### Western blot detection

20 μg protein samples were separated by 10% SDS-PAGE, transferred to polyvinylidene fluoride membrane, blocked in TBST containing 5% skim milk for 3 hours. Specific primary antibodies were added into the membrane overnight. Then the secondary antibody goat anti-rabbit IgG labeled with peroxidase were added. After one hour of incubation, the bands were analyzed with a gel imaging system. Antibodies were shown in Table 1.

**Table 1:** Antibodies used in western blot.

**Name**	**Brand**	**Source**	**Proportion**	**Clonality**	**Cat**
GAPDH antibody	Abcam	Mouse	1:10000	Monoclonal	ab8245
α-SMA antibody	Cell Signaling Technology	Rabbit	1:1000	Monoclonal	19245T
vimentin antibody	Abcam	Rabbit	1:1000	Monoclonal	ab92547
collagen I antibody	Abcam	Rabbit	1:1000	Monoclonal	ab6586
collagen III antibody	Abcam	Rabbit	1:1000	Monoclonal	ab184993
Klf4 antibody	Abcam	Rabbit	1:1000	Monoclonal	ab215036
STAT3 antibody	Abcam	Rabbit	1:1000	Monoclonal	ab68153
STAT3 (phospho Y705) antibody	Abcam	Rabbit	1:1000	Monoclonal	ab76315
Smad2/3 antibody	Abcam	Rabbit	1:1000	Monoclonal	ab202445
Smad2/3 (phospho T8) antibody	Abcam	Rabbit	1:1000	Monoclonal	ab272332
p38 MAPK antibody	Cell Signaling Technology	Rabbit	1:1000	Monoclonal	8690S
TGFβ1 receptor II antibody	Abcam	Rabbit	1:1000	Monoclonal	ab184948

### qRT-PCR

Total RNAs were extracted from mice lung tissues or L929 cells. 1 μg RNA was reversely transcribed into cDNA by using one-step RT-PCR kit (TaKaRa Biotechnology). After cDNA obtainment, the PCR reaction was carried out in a 20 μL system including 500 ng/μL cDNA 2 μL, 0.4 μL reverse and forward primers, 10 μL SYBR and 7.2 μL DEPC water. The reaction procedure was as follows: the initial denaturation at 95° C for 30 seconds, followed by 45 cycles of 95° C for 5 seconds, 60° C for 15 seconds and the dissolution curve stage at 95° C for 15 seconds, 60° C for 1 minute, 95° C for 1 second. GAPDH was used as an internal standard and results were calculated by using 2-ΔΔCT method. Primers as the follows:

lnc865: 5’-GGGAAAGGAATTAAGGCTCTCA-3’ and 5’-ACAGTGCATGTCTCCAGTTGTATCT-3’; lnc556: 5’-GACTGGCTGGTATTCTTTATTGCA-3’ and 5’-GCTTAAAAGGACACAACTGGGA-3’.

### HE and Masson's trichrome staining

Lung tissues were fixed with 4% paraformaldehyde overnight, dehydrated by an automatic dehydrator, and embedded with paraffin. After slicing, 4 μM sections were stained with HE or modified Masson’s trichrome stain Kit. Visual fields were randomly chosen to obtain under a microscope.

### Whole transcriptome sequencing

RNA samples were extracted from sham group, BLM group and AOBEE + BLM group, respectively. Their qualities were detected by agarose gel electrophoresis. LncRNAs were further purified from total RNA using magnetic beads attached to poly-T oligonucleotides to remove other RNA. NEBNext® Ultra™ RNA library was used to generate a sequencing library according to the manufacturer’s recommendation. After quantization and standardization the raw data, lncRNAs with more than 2 tags were selected for further analysis. The differentially expressed lncRNAs were identified via folding change filter.

### CCK-8 toxicity test

L929 cells were seeded in a 96-well flat bottom at 2×10^4^ cells/well, and then incubated at 37° C under 5% CO_2_ for 24 h. L929 cells were divided into different groups. Each group had six repetitive holes. CCK-8 reagent and 100μL serum-containing medium were added to each well and incubated at 37° C for 4 h. A microplate reader detected the absorbance at 450 nm. The cell proliferation was calculated according to the following formula: cell proliferation (%) = (measurement tube absorbance-blank absorbance) / (standard tube absorbance-blank absorbance) × 100%.

### Degradation experiment

5 mg actidione was dissolved in 1 mL DMSO. Then, 1 uL dissolved actidione was added to 1000 uL culture medium. The culture medium containing actidione was added to cell samples every two hours. After 8 hours, cell samples were harvested.

### Statistical analysis

Statistical analysis was performed using SPSS version 25.0 software. The student’s t test was used to evaluate the difference in measurement variables between the experimental group and the control group. Data were presented as the mean ± SD of at least three independent experiments, and p < 0.05 was considered statistically significant.

## Supplementary Material

Supplementary Video 1

Supplementary Video 2

Supplementary Video 3

## References

[1] Xue M, Guo Z, Cai C, Sun B, Wang H. Evaluation of the diagnostic efficacies of serological markers KL-6, SP-A, SP-D, CCL2, and CXCL13 in idiopathic interstitial pneumonia. Respiration. 2019; 98:534–45. 10.1159/000503689 31665737

[2] Meyer KC. Pulmonary fibrosis, part I: epidemiology, pathogenesis, and diagnosis. Expert Rev Respir Med. 2017; 11:343–59. 10.1080/17476348.2017.1312346 28345383

[3] Noble PW, Albera C, Bradford WZ, Costabel U, Glassberg MK, Kardatzke D, King TE Jr, Lancaster L, Sahn SA, Szwarcberg J, Valeyre D, du Bois RM, and CAPACITY Study Group. Pirfenidone in patients with idiopathic pulmonary fibrosis (CAPACITY): two randomised trials. Lancet. 2011; 377:1760–69. 10.1016/S0140-6736(11)60405-4 21571362

[4] Flaherty KR, Wells AU, Cottin V, Devaraj A, Walsh SL, Inoue Y, Richeldi L, Kolb M, Tetzlaff K, Stowasser S, Coeck C, Clerisme-Beaty E, Rosenstock B, et al, and INBUILD Trial Investigators. Nintedanib in progressive fibrosing interstitial lung diseases. N Engl J Med. 2019; 381:1718–27. 10.1056/NEJMoa1908681 31566307

[5] Gil N, Ulitsky I. Regulation of gene expression by cis-acting long non-coding RNAs. Nat Rev Genet. 2020; 21:102–17. 10.1038/s41576-019-0184-5 31729473

[6] Song X, Xu P, Meng C, Song C, Blackwell TS, Li R, Li H, Zhang J, Lv C. lncITPF promotes pulmonary fibrosis by targeting hnRNP-L depending on its host gene ITGBL1. Mol Ther. 2019; 27:380–93. 10.1016/j.ymthe.2018.08.026 30528088 PMC6369732

[7] Chen H, Wang J, Li R, Lv C, Xu P, Wang Y, Song X, Zhang J. Astaxanthin attenuates pulmonary fibrosis through lncITPF and mitochondria-mediated signal pathways. J Cell Mol Med. 2020; 24:10245–50. 10.1111/jcmm.15477 32813323 PMC7520307

[8] Viereck J, Bührke A, Foinquinos A, Chatterjee S, Kleeberger JA, Xiao K, Janssen-Peters H, Batkai S, Ramanujam D, Kraft T, Cebotari S, Gueler F, Beyer AM, et al. Targeting muscle-enriched long non-coding RNA H19 reverses pathological cardiac hypertrophy. Eur Heart J. 2020; 41:3462–74. 10.1093/eurheartj/ehaa519 32657324 PMC8482849

[9] Qian W, Cai X, Qian Q, Wang D, Zhang L. Angelica sinensis polysaccharide suppresses epithelial-mesenchymal transition and pulmonary fibrosis via a DANCR/AUF-1/FOXO3 regulatory axis. Aging Dis. 2020; 11:17–30. 10.14336/AD.2019.0512 32010478 PMC6961774

[10] Qian W, Cai X, Qian Q. Sirt1 antisense long non-coding RNA attenuates pulmonary fibrosis through sirt1-mediated epithelial-mesenchymal transition. Aging (Albany NY). 2020; 12:4322–36. 10.18632/aging.102882 32139663 PMC7093192

[11] Zhang J, Chen X, Chen H, Li R, Xu P, Lv C, Liu B, Song X. Engeletin ameliorates pulmonary fibrosis through endoplasmic reticulum stress depending on lnc949-mediated TGF-β1-Smad2/3 and JNK signalling pathways. Pharm Biol. 2020; 58:1105–14. 10.1080/13880209.2020.1834590 33181025 PMC7671710

[12] Li C, Wang Z, Zhang J, Zhao X, Xu P, Liu X, Li M, Lv C, Song X. Crosstalk of mRNA, miRNA, lncRNA, and circRNA and their regulatory pattern in pulmonary fibrosis. Mol Ther Nucleic Acids. 2019; 18:204–18. 10.1016/j.omtn.2019.08.018 31561125 PMC6796619

[13] Zhang G, Li S, Lu J, Ge Y, Wang Q, Ma G, Zhao Q, Wu D, Gong W, Du M, Chu H, Wang M, Zhang A, Zhang Z. LncRNA MT1JP functions as a ceRNA in regulating FBXW7 through competitively binding to miR-92a-3p in gastric cancer. Mol Cancer. 2018; 17:87. 10.1186/s12943-018-0829-6 29720189 PMC5930724

[14] Xie C, Yuan J, Li H, Li M, Zhao G, Bu D, Zhu W, Wu W, Chen R, Zhao Y. NONCODEv4: exploring the world of long non-coding RNA genes. Nucleic Acids Res. 2014; 42:D98–103. 10.1093/nar/gkt1222 24285305 PMC3965073

[15] Huang Y. The novel regulatory role of lncRNA-miRNA-mRNA axis in cardiovascular diseases. J Cell Mol Med. 2018; 22:5768–75. 10.1111/jcmm.13866 30188595 PMC6237607

[16] Zhao H, Shi J, Zhang Y, Xie A, Yu L, Zhang C, Lei J, Xu H, Leng Z, Li T, Huang W, Lin S, Wang L, et al. LncTarD: a manually-curated database of experimentally-supported functional lncRNA-target regulations in human diseases. Nucleic Acids Res. 2020; 48:D118–26. 10.1093/nar/gkz985 31713618 PMC7145524

[17] Janaki Ramaiah M, Divyapriya K, Kartik Kumar S, Rajesh YB. Drug-induced modifications and modulations of microRNAs and long non-coding RNAs for future therapy against glioblastoma multiforme. Gene. 2020; 723:144126. 10.1016/j.gene.2019.144126 31589963

[18] Wang W, Jia YJ, Yang YL, Xue M, Zheng ZJ, Wang L, Xue YM. LncRNA GAS5 exacerbates renal tubular epithelial fibrosis by acting as a competing endogenous RNA of miR-96-5p. Biomed Pharmacother. 2020; 121:109411. 10.1016/j.biopha.2019.109411 31810140

[19] Gao M, Dong Z, Sun J, Liu W, Xu M, Li C, Zhu P, Yang X, Shang X, Wu Y, Liu S. Liver-derived exosome-laden lncRNA MT1DP aggravates cadmium-induced nephrotoxicity. Environ Pollut. 2020; 258:113717. 10.1016/j.envpol.2019.113717 31864927

[20] Xu W, Zhou G, Wang H, Liu Y, Chen B, Chen W, Lin C, Wu S, Gong A, Xu M. Circulating lncRNA SNHG11 as a novel biomarker for early diagnosis and prognosis of colorectal cancer. Int J Cancer. 2020; 146:2901–12. 10.1002/ijc.32747 31633800

[21] Qadir MI, Bukhat S, Rasul S, Manzoor H, Manzoor M. RNA therapeutics: identification of novel targets leading to drug discovery. J Cell Biochem. 2020; 121:898–929. 10.1002/jcb.29364 31478252

[22] Lü S, Liu Y, Cui J, Yang B, Li G, Guo Y, Kuang H, Wang Q. Mechanism of caulophyllum robustum maxim against rheumatoid arthritis using LncRNA-mRNA chip analysis. Gene. 2020; 722:144105. 10.1016/j.gene.2019.144105 31521702

[23] Wei LB, Gao JR, Gao YC, Liu XC, Jiang H, Qin XJ. Effect of the traditional Chinese medicine Qi Teng Xiao Zhuo granules on chronic glomerulonephritis rats studied by using long noncoding RNAs expression profiling. Gene. 2020; 728:144279. 10.1016/j.gene.2019.144279 31821871

[24] Wang C, Cao H, Gu S, Shi C, Chen X, Han X. Expression analysis of microRNAs and mRNAs in myofibroblast differentiation of lung resident mesenchymal stem cells. Differentiation. 2020; 112:10–16. 10.1016/j.diff.2019.11.002 31838455

[25] Liao X, Sharma N, Kapadia F, Zhou G, Lu Y, Hong H, Paruchuri K, Mahabeleshwar GH, Dalmas E, Venteclef N, Flask CA, Kim J, Doreian BW, et al. Krüppel-like factor 4 regulates macrophage polarization. J Clin Invest. 2011; 121:2736–49. 10.1172/JCI45444 21670502 PMC3223832

[26] Wen Y, Lu X, Ren J, Privratsky JR, Yang B, Rudemiller NP, Zhang J, Griffiths R, Jain MK, Nedospasov SA, Liu BC, Crowley SD. KLF4 in macrophages attenuates TNF α-mediated kidney injury and fibrosis. J Am Soc Nephrol. 2019; 30:1925–38. 10.1681/ASN.2019020111 31337692 PMC6779357

[27] Morin F, Kavian N, Chouzenoux S, Cerles O, Nicco C, Chéreau C, Batteux F. Leflunomide prevents ROS-induced systemic fibrosis in mice. Free Radic Biol Med. 2017; 108:192–203. 10.1016/j.freeradbiomed.2017.03.035 28365359

[28] Liu B, Li R, Zhang J, Meng C, Zhang J, Song X, Lv C. MicroRNA-708-3p as a potential therapeutic target via the ADAM17-GATA/STAT3 axis in idiopathic pulmonary fibrosis. Exp Mol Med. 2018; 50:e465. 10.1038/emm.2017.311 29869625 PMC5898903

[29] Bourillot PY, Aksoy I, Schreiber V, Wianny F, Schulz H, Hummel O, Hubner N, Savatier P. Novel STAT3 target genes exert distinct roles in the inhibition of mesoderm and endoderm differentiation in cooperation with nanog. Stem Cells. 2009; 27:1760–71. 10.1002/stem.110 19544440

[30] Kime C, Sakaki-Yumoto M, Goodrich L, Hayashi Y, Sami S, Derynck R, Asahi M, Panning B, Yamanaka S, Tomoda K. Autotaxin-mediated lipid signaling intersects with LIF and BMP signaling to promote the naive pluripotency transcription factor program. Proc Natl Acad Sci USA. 2016; 113:12478–83. 10.1073/pnas.1608564113 27738243 PMC5098653

[31] Moreira D, Zhang Q, Hossain DM, Nechaev S, Li H, Kowolik CM, D’Apuzzo M, Forman S, Jones J, Pal SK, Kortylewski M. TLR9 signaling through NF-κB/RELA and STAT3 promotes tumor-propagating potential of prostate cancer cells. Oncotarget. 2015; 6:17302–13. 10.18632/oncotarget.4029 26046794 PMC4627309

